# Sestrin 2 confers primary resistance to sorafenib by simultaneously activating AKT and AMPK in hepatocellular carcinoma

**DOI:** 10.1002/cam4.1826

**Published:** 2018-10-11

**Authors:** Jimin Dai, Qichao Huang, Kunwei Niu, Bo Wang, Yijie Li, Chen Dai, Zhinan Chen, Kaishan Tao, Jingyao Dai

**Affiliations:** ^1^ Department of Hepatobiliary Surgery The First Affiliated Hospital of Air Force Medical University Xi'an China; ^2^ The Cadet Team 6 (Regiment 6) of School of Basic Medicine Air Force Medical University Xi'an China; ^3^ State Key Laboratory of Cancer Biology and Experimental Teaching Center of Basic Medicine Air Force Medical University Xi'an China; ^4^ Department of Orthopedics The First Affiliated Hospital of Air Force Medical University Xi'an China; ^5^ Department of Cell Biology, National Translational Science Center for Molecular Medicine Air Force Medical University Xi'an China

**Keywords:** AKT, AMPK, hepatocellular carcinoma, primary resistance, Sestrin2, sorafenib

## Abstract

Hepatocellular carcinoma (HCC) is the malignancy derived from normal hepatocytes with increasing incidence and extremely poor prognosis worldwide. The only approved first‐line systematic treatment agent for HCC, sorafenib, is capable to effectively improve advanced HCC patients’ survival. However, it is gradually recognized that the therapeutic response to sorafenib could be drastically diminished after short‐term treatment, defined as primary resistance. The present study is aimed to explore the role of stress‐inducible protein Sestrin2 (SESN2), one of the most important sestrins family members, in sorafenib primary resistance. Herein, we initially found that SESN2 expression was significantly up‐regulated in both HCC cell lines and tissues compared to normal human hepatocytes and corresponding adjacent liver tissues, respectively. In addition, SESN2 expression was highly correlated with sorafenib IC_50_ of HCC cell lines. Thereafter, we showed that sorafenib treatment resulted in an increase of SESN2 expression and the knockdown of SESN2 exacerbated sorafenib‐induced proliferation inhibition and cell apoptosis. Further mechanistic study uncovered that SESN2 deficiency impaired both AKT and AMPK phosphorylation and activation after sorafenib treatment. Moreover, the correlations between SESN2 expression and both phosphor‐AKT and phosphor‐AMPK expression were illustrated in HCC tissues. Taken together, our study demonstrates that SESN2 activates AKT and AMPK signaling as a novel mechanism to induce sorafenib primary resistance in HCC.

## 1 INTRODUCTION

Hepatocellular carcinoma (HCC) is the third primary cause of cancer‐related death, accounting for nearly 745 000 deaths annually worldwide,^1^ and the 5‐year survival rate of HCC patients is no more than 40%.^2^ Sorafenib, the multi‐kinase inhibitor, is the first‐line systemic therapy agent approved by the Food and Drug Administration in 2008.^3^ As the inhibitor of Raf/MEK/extracellular signaling‐regulated kinase (Raf/MEK/ERK),^4^ sorafenib is able to suppress tumor growth and angiogenesis, thereby delaying HCC progression with the prolongation of the patients' survival for almost 3 months.^5^ Nevertheless, the efficacy of sorafenib is still confronted with several challenges. On one hand, decreased therapeutic response toward sorafenib has been widely acknowledged after long‐term medication with the occurrence like aberrant changes in the Janus kinase/signal transducer and activator of transcription (JAK/STAT) pathway,^6^ elevation of autophagy^7^, ^8^ and epithelial‐mesenchymal transition (EMT),^9^, ^10^ regarded as acquired resistance.^11^ On the other hand, initially impaired therapeutic efficacy after short‐term treatment is known as primary resistance, partly due to genetic heterogeneity.^12^ Additionally, the rapid inducible activation of intrinsic pro‐survival signaling pathways like phosphoinositide 3‐kinase/protein kinase B (PI3K/AKT)^13^, ^14^, ^15^ and cell growth‐associated signaling pathways like epidermal growth factor receptor/HER‐3 (EGFR/HER‐3)^16^, ^17^, ^18^ contributed to the impairment of sorafenib short‐term therapeutic effects.^19^ Notably, previous studies have mainly focused on the mechanism underlying sorafenib acquired resistance, whereas the mechanism of primary resistance demands further exploration. Thus, it is of necessity to reveal the potential mechanisms of sorafenib primary resistance to enhance the efficacy of short‐term sorafenib treatment and to improve HCC patient prognosis.

Sestrins are a crucial stress‐inducible protein family which is greatly implicated in maintaining cellular homeostasis. Sestrin2 (SESN2), one of the most important family members, has been reported to participate in tumorigenesis and tumor progression by regulating multiple downstream molecules, among which AKT and AMPK are tightly connected to cell proliferation and metabolism in tumor biology.^20^ To be specific, it has been revealed that AKT was involved in the facilitative effect of SESN2 on tumor progression by suppressing cell apoptosis in human prostate cancer.^21^ In addition, SESN2 was capable to induce resistance through activating AKT against vemurafenib in melanoma and against 5‐fluorouracil (5‐FU) in squamous cell carcinoma, respectively.^13^ Moreover, SESN2 was ascribed to promoting the survival and proliferation of ovarian cancer cells by suppressing cytotoxicity of natural killer cells via AMPK.^22^ Thus, SESN2 has exerted its pivotal role in mediating tumor progression via AKT and AMPK. To date, several lines of evidence showed contradictive pathogenic role of SESN2 in HCC. Chen et al^23^ reported that SESN2 expression markedly declined in HCC tissues in comparison to normal liver tissues, indicating its tumor suppressive role. In contrast, Buitrago‐Molina et al^24^ found that SESN2 compensated loss of the cyclin‐dependent kinase inhibitor p21gene as the oncogenic factor to promote HCC development via Nrf2 in mice. However, whether SESN2 expression is altered after sorafenib treatment and involved in sorafenib primary resistance in HCC remains to be unveiled. Given that SESN2 is greatly implicated in protecting cancer cells from apoptosis and promoting cell survival via AKT and AMPK, we hypothesized that it might confer primary resistance to sorafenib by activating AKT and AMPK in HCC.

In this study, we found that SESN2 expression was significantly up‐regulated in both HCC cells and tissues at first, and the increased expression of SESN2 was markedly correlated with sorafenib sensitivity of HCC cells. Subsequently, we showed that SESN2 expression was prominently induced after sorafenib treatment, and SESN2 deficiency forwardly impaired cellular proliferation and increased apoptosis by sorafenib. Mechanistically, the knockdown of SESN2 attenuated sorafenib‐induced activation of pro‐survival AKT and AMPK signaling pathways in HCC cells. What is more, the expression of SESN2 was highly associated with both phosphor‐AKT and phosphor‐AMPK in HCC tissues, indicating the involvement of SESN2 in triggering activation of AKT and AMPK in vivo. Collectively, our findings demonstrate that SESN2 activates AKT and AMPK signaling as a novel mechanism to induce sorafenib primary resistance in HCC cells.

## 2 MATERIALS AND METHODS

### 2.1 Cell culture and reagents

Normal human hepatic cell line LO2 and human hepatocellular carcinoma cell lines Bel‐7404, SNU‐368, HLE, HLF, and Hep3B were purchased from ATCC (Manassas, VA, USA). All these HCC cell lines were authenticated by short‐tandem repeat (STR) DNA testing by the Air Force Medical University Center for DNA Typing and tested for mycoplasma contamination. Bel‐7404 and SNU‐368 were cultured in RPMI 1640 (Invitrogen, Carlsbad, CA, USA) containing 10% fetal bovine serum (FBS, Invitrogen). LO2, HLE, HLF, and Hep3B were cultured in Dulbecco's modified Eagle medium (DMEM, Invitrogen) containing 10% FBS. All cells were incubated in humidified atmosphere of 5% CO_2_ at 37°C. DMSO was purchased from Sigma‐Aldrich (St. Louis, MO). Sorafenib was purchased from Bayer (Leverkusen, Germany).

### 2.2 Clinical specimens

Hepatocellular carcinoma and corresponding adjacent liver tissue specimens for immunohistochemical staining were collected from 30 HCC patients who received HCC resection from December 2017 to March 2018 in the Department of Hepatobiliary Surgery of the First Affiliated Hospital of Air Force Medical University, and the specimens were histologically confirmed as HCC by the Department of Pathology of the First Affiliated Hospital of Air Force Medical University. All patients were informed consent and informed consent forms were obtained from the involved patients. The research protocol, approved by the Ethical Committee of Air Force Medical University, was designed and executed according to the principles of the Declaration of Helsinki and its later amendments or comparable ethical standards.

### 2.3 Cell proliferation assay

Bel‐7404, SNU‐368, HLE, HLF, and Hep3B HCC cells were seeded in 96‐well plates with 5.0 × 10^3^ cells in each well, and then incubated in 5% CO_2_ at 37°C overnight. After that, the cells were cultured in the medium with (0, 2, 5, 10, 15, 20, and 25) μmol/L sorafenib for 24 hours. To examine the proliferation rates of HCC cell lines after sorafenib treatment, cell counting kit‐8 (CCK‐8) (EnoGene, Nanjing, China) was employed according to the manufacturer's protocol. In brief, the CCK‐8 reagent was added to each culture well and the cells were incubated at 37°C for 1 hour. Absorbance at 450 nm (A_450_) was detected with an Epoch Microplate Spectrophotometer (BioTek Instruments, Inc, Winooski, VT, USA). 50% inhibitory concentration (IC_50_) was calculated using GraphPad Prism 6.0 as previously described.^25^

### 2.4 Immunoblotting and antibodies

Bel‐7404 and SNU‐368 cell lines were cultured in the medium with (0, 2, 4, 6, and 8) μmol/L sorafenib for 24 hours, respectively, before they were lysed in RIPA buffer (Beyotime Biotechnology, Jiangsu, China) added with PMSF (Beyotime). Protein concentration was measured using the bicinchoninic acid (BCA) method kit (Solarbio, Beijing, China). Protein samples were separated by 10% SDS‐PAGE (Beyotime) and then transferred to polyvinylidene fluoride membranes (PVDF, Millipore, MA, USA). After blocking with 5% non‐fat milk for 1 hour, the membrane was incubated with primary antibodies at 4°C overnight and with the corresponding horse radish peroxidase (HRP)‐conjugated secondary antibody (1:2000 dilution) for 1 hour at room temperature the next day. Finally, the blots were detected using enhanced chemiluminescence substrate (ECL kit, Millipore). The phosphorylated protein was normalized to the corresponding total protein. The primary antibodies used for immunoblotting were against SESN2 (rabbit polyclonal, ProteinTech #10795‐1‐AP, diluted with 1:1000), AMPKα1 (rabbit polyclonal, ProteinTech #10929‐2‐AP, diluted with 1:300), Bcl‐2 (rabbit polyclonal, ProteinTech #12789‐1‐AP, diluted with 1:1000), Bax (rabbit polyclonal, ProteinTech #50599‐2‐Ig, diluted with 1:2000), GAPDH (rabbit monoclonal, Cell Signaling Technology, Danvers, MA, USA; #5174, diluted with 1:1000), AKT (rabbit monoclonal, Cell Signaling Technology #4685, diluted with 1:1000), phosphor‐AKT (Ser473) (p‐AKT (Ser473), rabbit monoclonal, Cell Signaling Technology #4060, diluted with 1:1000), phosphor‐AMPKα (Thr172) (p‐AMPKα (Thr172), rabbit monoclonal, Cell Signaling Technology #2535, diluted with 1:1000), cleaved‐caspase3 (rabbit monoclonal, Cell Signaling Technology #9664, diluted with 1:1000) and the second antibody was HRP‐linked antibody (goat anti‐rabbit IgG, Cell Signaling Technology #7074, diluted with 1:2000).

### 2.5 RNA extraction and qRT‐PCR

Total RNA was extracted using TRIzol reagent (Invitrogen) according to the manufacturer's instructions. Reverse transcription was performed using PrimeScript RTase (Takara Bio Inc, Tokyo, Japan) according to the manufacturer's protocol. The expression levels of SESN2 mRNA in Bel‐7404 and SNU‐368 HCC cell lines were determined with real‐time quantitative reverse transcription PCR (qRT‐PCR) using Premix Ex Taq (Takara) according to the manufacturer's instructions and normalized to the expression levels of the endogenous control, β‐actin. The cycling conditions were as follows: 95°C for 2 minutes followed by 40 cycles of denaturation at 95°C for 5 seconds, annealing at 55°C for 10 seconds, and extension at 72°C for 45 seconds. All reactions were run in triplicate. The resulting amplification and melt curves were analyzed to ensure the identity of the specific PCR product. Threshold cycle values were used to calculate the fold change in the transcript levels by using the 2^−ΔΔ^ *^Ct^* method. The primers used for qRT‐PCR were: β‐actin: sense 5′‐ GAGACCTTCAACACCCAGCC ‐3′, and antisense 5′‐TGCCATGGGTGGAATCATATTGG‐3′; SESN2: sense 5′‐ CTCACACCATTAAGCATGGAG ‐3′ and antisense 5′‐ CAAGCTCGGAATTAATGTGCC ‐3′.

### 2.6 Immunohistochemical (IHC) staining and analysis

For IHC staining, the clinical specimens were fixed with paraformaldehyde, embedded with paraffin and sectioned into 4‐μm‐thick slices. The subsequent steps accomplished with biotin‐streptavidin peroxidase method (SPlink Detection Kit, ZSGB‐Bio, Beijing, China) were followed according to the manufacturer's instructions. In brief, the paraffin‐embedded slides were deparaffinized, rehydrated with graded ethanol dilutions, subjected to antigen retrieval and blocked with H_2_O_2_ and goat serum. The slides were incubated with the corresponding primary antibodies at 4°C overnight. Then, the slides were washed with PBS, followed by incubated with biotinylated goat anti‐rabbit IgG and then incubated with HRP‐conjugated streptomycin. Diaminobenzidine (ZSGB‐Bio) was added to the slides for chromogenic reaction. The slides were mounted and observed with optical microscope (Olympus, Tokyo, Japan). The primary antibodies used for IHC staining were against SESN2 (ProteinTech #10795‐1‐AP, diluted with 1:200), Ki‐67 (Cell Signaling Technology #9027, diluted with 1:400), phosphor‐AKT (Ser473) (Cell Signaling Technology #4060, diluted with 1:100), phosphor‐AMPKα (Thr172) (Cell Signaling Technology #2535, diluted with 1:100).

The standard of staining scores was described previously.^26^ In brief, the percentages of staining‐positive cells were evaluated into four categories: 0 (0%), 1 (1%‐33%), 2 (34%‐66%), and 3 (67%‐100%). The staining intensities were evaluated into four grades: 0 (none), 1 (week), 2 (moderate), and 3 (strong). The final staining score was defined as the product of the percentage and intensity scores.

### 2.7 RNA interference

Small interfering RNA (siRNA) specifically targeting SESN2 (siSESN2) and negative control siRNA (siNC) were designed and synthesized by GenePharm (Shanghai, China). Sequences of the siRNA were as follows: siSESN2: sense 5′‐GAAGACCCTACTTTCGGAT‐3′, antisense 5′‐ATCCGAAAGTAGGGTCTTC‐3′; siNC: sense 5′‐UUCUCCGAACGUGUCACGUTT‐3′, antisense 5′‐ACGUGACACGUUCGGAGAATT‐3′. The siRNA was transfected into cells using Lipofectamine^TM^ 2000 (Invitrogen), and the transfection procedures were performed according to the manufacturer's instructions.

### 2.8 Flow cytometry for cell apoptosis analysis

Bel‐7404 and SNU‐368 cells transfected with siNC RNA or siSESN2 RNA as described above were seeded in 6‐well plates at the density of 2.5 × 10^5^ cells per well and were then incubated with 8 μmol/L sorafenib for 24 hours. The cells were harvested by trypsinization (Solarbio) and washed twice with 4°C PBS. Before apoptosis analysis by flow cytometry (Beckman Coulter, Miami, FL), the cells were stained with annexin V‐FITC/PI (Annexin V‐FITC/PI Apoptosis Detection Kit, Beyotime) according to the manufacturer's instructions.

### 2.9 Detection of intracellular ATP levels

Enhanced ATP Assay Kit (Beyotime) by luciferin‐luciferase method was used to measure ATP levels in the transfected Bel‐7404 and SNU‐368 cells with/without sorafenib treatment. After indicated treatment, the cells were lysed and ATP concentration standard curves were calculated according to the manufacturer's instructions. The luminometer, Fluoroskan Ascent FL (Thermo Scientific, Waltham, MA, USA) was utilized for fluorescence detection released from luciferin.

### 2.10 Statistical analysis

The results were analyzed by two‐tailed Student's *t* test. Spearman correlation analysis and linear regression analysis were used to assess the correlation between SESN2 relative expression levels and IC_50_ values of HCC cell lines and the correlations among SESN2 IHC scores and those of phosphor‐AKT (Ser473), phosphor‐AMPKα (Thr172), respectively. The results were presented as Mean ± SD through at least three independent experiments, with *P < *0.05 considered to be statistically significant. Except for IC_50_ calculation by GraphPad Prism 6.0 (GraphPad Software, La Jolla, CA, USA), other statistical analyses were accomplished using SPSS 17.0 (SPSS Inc, Chicago, IL, USA).

## 3 RESULTS

### 3.1 SESN2 expression is significantly up‐regulated in both HCC cells and tissues

**Figure 1:**
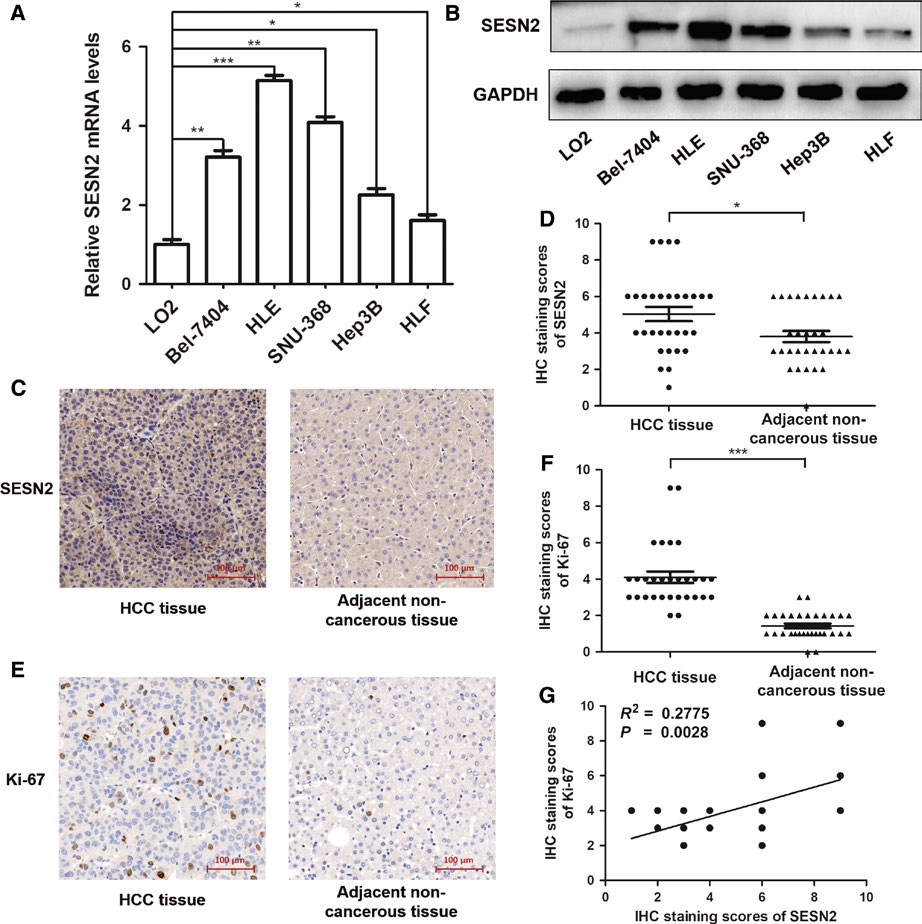
SESN2 was significantly up‐regulated in HCC cell lines and tissues. (A) qRT‐PCR and (B) immunoblotting analysis of SESN2 expression levels in HCC cell lines and normal hepatocytes. (C and E) Representative images of SESN2 and Ki‐67 expression by IHC staining analysis in HCC tissues and the corresponding adjacent liver tissues. Scale bar = 100 μm. (D and F) The IHC staining scores of SESN2 and Ki‐67 in HCC tissues and the corresponding adjacent liver tissues (n = 30). (G) Correlation between SESN2 IHC staining scores and Ki‐67 IHC staining scores of HCC tissues was tested by Spearman's correlation analysis (*R* ^2 ^= 0.2775, ^**^ *P* = 0.0028) (n = 30, cases sharing the identical IHC scores overlap in the graph). The results are presented as mean ± SD through at least three independent experiments and are analyzed by two‐tailed Student's *t* test. ^*^ *P* < 0.05, ^**^ *P* < 0.01, ^***^ *P* < 0.001

To investigate the role of SESN2 in HCC, we firstly examined the expression of SESN2 in HCC cell lines and tissues. Our qRT‐PCR assay showed that compared with normal human hepatocytes (LO2), SESN2 mRNA levels were markedly higher in a panel of HCC cell lines, including Bel‐7404, HLF, HLE, SNU‐368, and Hep3B (Figure 1A). Consistent with this, SESN2 protein levels were also significantly up‐regulated in HCC cell lines (Figure 1B). Further, we employed IHC staining analysis in HCC tissues and corresponding adjacent liver tissues to detect SESN2 expression, the results of which were in a similar alteration trend as those in HCC cells (Figure 1C,D). More importantly, we determined the correlation between expression levels of SESN2 and cell proliferation biomarker, Ki‐67, which was also drastically increased in HCC tissues (Figure 1E,F). As a result, we revealed that SESN2 expression was remarkably correlated with Ki‐67 expression (Figure 1G), demonstrating the great potential of SESN2 as a valuable biomarker in determining HCC progression and prognosis. Taken together, these data illustrated that SESN2 expression was significantly up‐regulated in HCC, suggesting that it could be endowed with a potential tumorigenic effect.

### 3.2 SESN2 expression is positively correlated with IC_50_ of sorafenib in HCC cell lines

**Figure 2:**
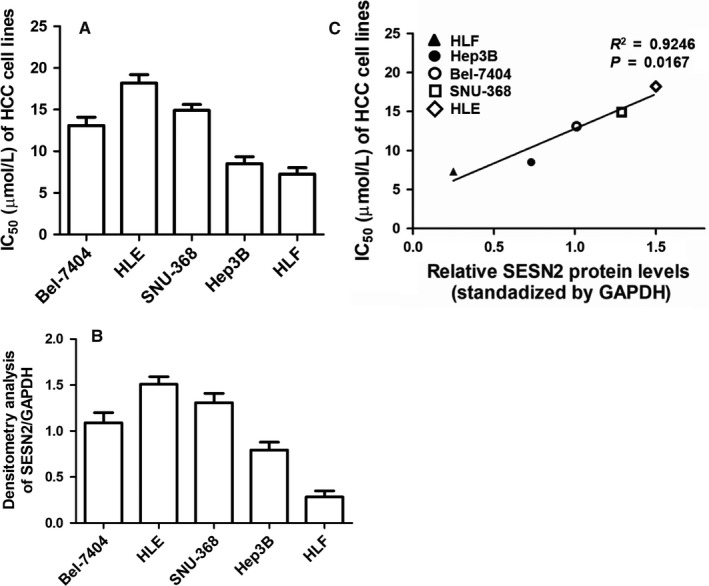
SESN2 expression was positively associated with sorafenib IC_50_ values of HCC cell lines_._ (A) IC_50_ values of sorafenib and (B) densitometry analysis for SESN2 expression from immunoblotting results of the HCC cell lines. (C) Positive correlation between SESN2 expression levels and IC_50_ in HCC cell lines by Spearman correlation analysis (*R* ^2 ^= 0.9246, ^*^ *P* = 0.0167). The results are presented as mean ± SD through at least three independent experiments

The promising systemic agent sorafenib merely demonstrated limited benefits to about 30% HCC patients since some patients are initially resistant to sorafenib.^27^ To testify the potential involvement of SESN2 in sorafenib therapeutic response, we first examined sorafenib IC_50_ of HCC cell lines including Bel‐7404, HLF, HLE, SNU‐368, and Hep3B by culturing them with 0, 2, 5, 10, 15, 20, and 25 μmol/L sorafenib, respectively, for 24 hours and then using CCK‐8 assay to examine cell viability. As was shown in the cell proliferation curves (Figure S1) and further analyses , the values of IC_50_ varied a lot among these different HCC cell lines (Figure 2A). In accordance with previous studies,^28^, ^29^ HLF exhibited a sorafenib‐sensitive characteristic while HLE was intrinsically sorafenib‐tolerant with the IC_50_ value over 15 μmol/L. Besides, the IC_50_ value of Hep3B was within 6‐8 μmol/L similar to previous studies,^29^, ^30^ while those of Bel‐7404 and SNU‐368 were between 13 and 15 μmol/L. Subsequently, we examined the correlation between SESN2 expression and sorafenib IC_50_ among HCC cell lines via Spearman correlation analysis. Interestingly, we found that the higher SESN2 expressed in HCC cell line (Figure 2B), the higher IC_50_ of the corresponding HCC cell line was. In particular, there was a prominently positive correlation between SESN2 expression levels and IC_50_ values of the indicated HCC cell lines (Figure 2C). Thus, it was of high possibility that SESN2 was greatly implicated in mediating the efficacy of short‐term sorafenib treatment.

### 3.3 SESN2 up‐regulation contributes to sorafenib primary resistance in HCC

**Figure 3:**
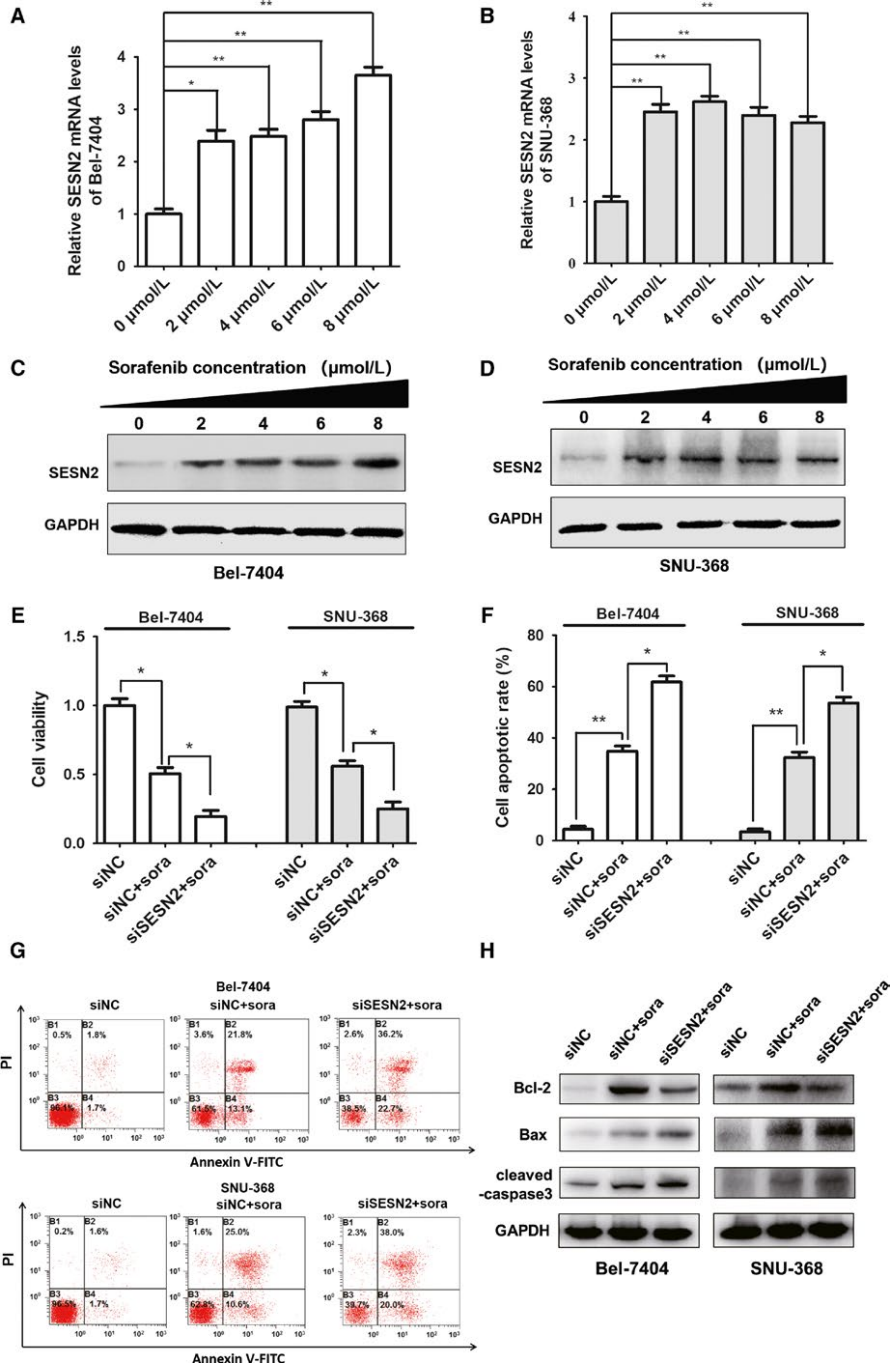
SESN2 up‐regulation contributed to sorafenib primary resistance in HCC. (A and B) qRT‐PCR analysis of SESN2 mRNA expression after sorafenib treatment with indicated concentrations in both Bel‐7404 and SNU‐368 HCC cells; (C and D) Immunoblotting analysis of SESN2 protein expression after sorafenib treatment with indicated concentrations in both Bel‐7404 and SNU‐368 HCC cells; (E) Cell viability of HCC cells with or without SESN2 knockdown after sorafenib treatment; (F) Flow cytometry analysis of HCC cell apoptosis with or without SESN2 knockdown after sorafenib treatment; (G) Representative flow cytometry images of cell apoptosis with or without SESN2 knockdown after sorafenib treatment; (H) Immunoblotting analysis of pro‐apoptotic Bax and cleaved‐caspase 3 together with anti‐apoptotic Bcl‐2 expression with or without SESN2 knockdown after sorafenib treatment. The results are presented as mean ± SD through at least three independent experiments and are analyzed by two‐tailed Student's *t* test. ^*^ *P* < 0.05, ^**^ *P* < 0.01

To explore the effects of SESN2 on the efficacy of short‐term sorafenib treatment, we selected Bel‐7404 and SNU‐368 HCC cell lines with relative low endogenous SESN2 expression for further studies. The two cell lines were cultured with 0, 2, 4, 6, and 8 μmol/L sorafenib for 24 hours, respectively. Our qRT‐PCR (Figure 3A,B) and immunoblotting assays (Figure 3C,D) showed significant elevation of both SESN2 mRNA and protein levels after this short‐term sorafenib administration in both cell lines which indicated that short‐term sorafenib treatment was capable to induce the increase of SESN2 expression.

Thereafter, in order to observe the biological influence of elevated SESN2 expression on sorafenib treatment efficiency, we used specific siRNA against SESN2 to obtain the knockdown of SESN2 in both Bel‐7404 and SNU‐368 cell lines, and then processed to short‐term sorafenib treatment for 24 hours. We conducted CCK8 assay to measure cell viability, and it revealed that in addition to impaired cell viability after sorafenib treatment, the knockdown of SESN2 led to further attenuation of cell viability in both cell lines (Figure 3E). Moreover, we performed flow cytometry assay to detect the alteration of cell apoptosis after the indicated treatment mentioned above. Consistently, while sorafenib mono‐treatment resulted in prominent increase of apoptotic cells, the concurrent knockdown of SESN2 forwardly aggravated the death of HCC cells (Figure 3F,G). Meanwhile, our immunoblotting analysis showed that SESN2 deficiency promoted the expression of pro‐apoptotic Bax and cleaved‐caspase 3, whereas inhibited the expression of anti‐apoptotic Bcl‐2 (Figure 3H). Taken together, our results showed that sorafenib administration up‐regulated SESN2 expression in both Bel‐7404 and SNU‐368 HCC cell lines and the knockdown of SESN2 not only reduced HCC cell viability but also promoted apoptosis of the HCC cells, demonstrating that SESN2 up‐regulation contributed to sorafenib primary resistance in HCC.

### 3.4 The knockdown of SESN2 abolishes the activation of AKT and AMPK after sorafenib treatment in HCC cell lines

**Figure 4:**
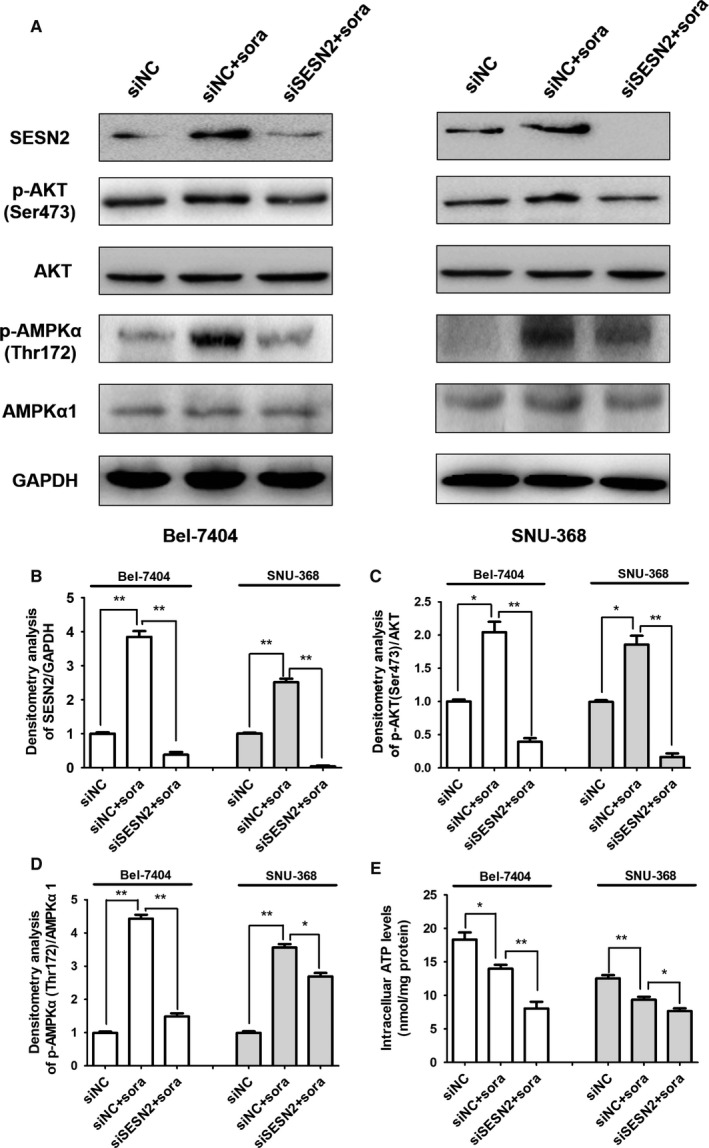
Knockdown of SESN2 inhibited activation of AKT and AMPK signaling after sorafenib administration in HCC cells. (A) Immunoblotting analysis of SESN2, AKT, phosphor‐AKT (Ser473), AMPKα1, phosphor‐AMPKα (Thr172) with or without SESN2 knockdown after sorafenib treatment in Bel‐7404 and SNU‐368 HCC cells. (B, C and D) Densitometry analysis for SESN2/GAPDH, phosphor‐AKT (Ser473)/AKT, and phosphor‐AMPKα (Thr172)/AMPKα1. (E) Intracellular ATP determination assay with or without SESN2 knockdown in Bel‐7404 and SNU‐368 HCC cells. The results are presented as mean ± SD through at least three independent experiments and are analyzed by two‐tailed Student's *t* test. ^*^ *P* < 0.05, ^**^ *P* < 0.01

As has been reported in prostate cancer, melanoma and squamous carcinoma,^13^, ^21^ SESN2 serves as an upstream regulator in AKT signaling pathway to maintain cancer cell survival. Therefore, we speculated that SESN2 up‐regulation might induce sorafenib primary resistance via activating AKT in HCC cells. To this end, we conducted immunoblotting analysis to examine the alteration of AKT and phosphor‐AKT (Ser473) expression in HCC cell lines with or without SESN2 knockdown after sorafenib administration. As was shown, the expression levels of SESN2 were successfully reduced after siRNA transfection in both cell lines (Figure 4A,B). More importantly, although sorafenib treatment induced prominent increase of phosphor‐AKT (Ser473), the silence of SESN2 markedly re‐suppressed the activation of AKT signaling (Figure 4A,C), supporting the notion that sorafenib treatment promoted AKT activation via the up‐regulation of SESN2 expression. Therefore, SESN2 was essential for activating AKT to further constrain cell apoptosis.

Aside from AKT signaling, AMPK is greatly involved in regulating ATP generation after energy deprivation, the stressful condition that can be induced by sorafenib treatment.^31^ Of note, it has been already revealed that SESN2 was responsible for AMPK activation to facilitate autophagy in protecting HCC cells from death.^32^ Hence, we proposed that SESN2 might mediate AMPK to maintain HCC cell survival after sorafenib treatment. We detected expression levels of AMPKα1 and phosphor‐AMPKα (Thr172) by immunoblotting analysis in the two HCC cell lines. Similarly, while sorafenib treatment significantly activated AMPK, the knockdown of SESN2 reversed the promotive influence of sorafenib on AMPK signaling (Figure 4A,D). In addition, we employed ATP determination assay and found that with the decline of ATP levels after sorafenib stimulation, the silence of SESN2 was capable to further attenuate the intracellular ATP levels (Figure 4E), suggesting that SESN2 deficiency blocked ATP generation by suppressing AMPK activation. Collectively, we revealed that SESN2 deficiency diminished the activation of AKT and AMPK signaling after sorafenib treatment, probably accounting for the inhibition of cell viability, the increase of cell apoptosis and concurrent reduction of ATP levels in HCC cells. In summary, SESN2 up‐regulation could facilitate sorafenib primary resistance.

### 3.5 SESN2 expression is positively correlated with the phosphorylation of both AKT and AMPK in HCC tissues

**Figure 5:**
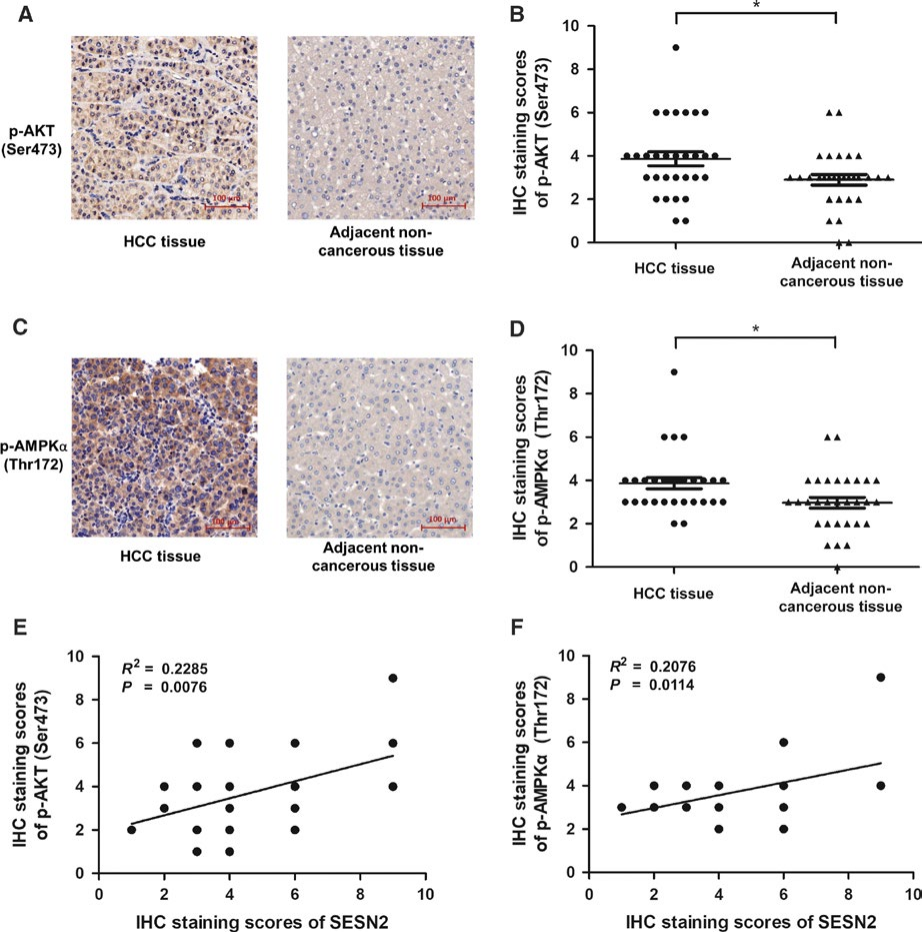
SESN2 expression was positively correlated with phosphorylation of AKT and AMPK in HCC tissues. (A and C) Representative images of phosphor‐AKT (Ser473) and phosphor‐AMPKα (Thr172) expression by IHC staining analysis in HCC tissues and the corresponding adjacent liver tissues. Scale bar = 100 μm. (B and D) The IHC staining scores of phosphor‐AKT (Ser473) and phosphor‐AMPKα (Thr172) in HCC tissues (n = 30). (E and F) Positive correlations between SESN2 expression levels and phosphor‐AKT (Ser473) (*R* ^2 ^= 0.2285, ^**^ *P* = 0.0076), phosphor‐AMPKα (Thr172) (*R* ^2 ^= 0.2076, ^*^ *P* = 0.0114), respectively, shown in the HCC tissues by Spearman correlation analysis (n = 30, cases sharing the identical IHC scores overlap in the graph). The results are presented as mean ± SD and are analyzed by two‐tailed Student's *t* test. *P<.05.

Finally, we wondered the relationships between SESN2 expression and phosphorylation of both AMPK and AKT in HCC tissues. Several lines of evidence showed the activation of AKT^33^, ^34^ or AMPK^35^, ^36^ mediated by multiple regulators in HCC cells, but the status of AKT and AMPK signaling pathway in HCC tissues has not been fully elucidated. To this end, we conducted IHC staining assay to examine the phosphorylation of AMPK and AKT in HCC tissues and corresponding adjacent noncancerous liver tissues. As was revealed, the phosphorylation of AKT was markedly increased in HCC tissues compared with adjacent noncancerous tissues (Figure 5A,B). Moreover, the staining score of phosphor‐AMPKα (Thr172) showed a similar alteration pattern as phosphor‐AKT (Ser473) (Figure 5C,D), thus indicating the activation of the two pathways in HCC tissues. Thereafter, we turned to analyze the association between SESN2 expression and phosphor‐AKT (Ser473), phosphor‐AMPKα (Thr172) expression, respectively, in HCC tissues. Our data showed a significantly positive correlation between SESN2 expression and the phosphorylation of AKT (Figure 5E). Aside from this, we proved that SESN2 expression was also highly associated with phosphor‐AMPKα (Thr172) (Figure 5F). Following these results, we demonstrated the close relationships between SESN2 expression and the activation of both AMPK and AKT pathways in HCC and more importantly, implying the involvement of SESN2 in the regulation of AMPK and AKT signaling in vivo.

## 4 DISCUSSION

In the present study, we found that the expression of stress‐inducible protein SESN2 was drastically increased in both HCC tissues and cell lines at first. Besides, SESN2 expression was in positive correlation with sorafenib IC_50_ in HCC cell lines. Subsequently, we uncovered that short‐term sorafenib treatment induced up‐regulation of SESN2 expression, and SESN2 knockdown aggravated sorafenib‐induced cell viability inhibition as well as cell apoptosis induction. Further, our mechanistic studies showed that SESN2 was capable to activate both AKT and AMPK pathways, potentially conferring primary resistance to sorafenib treatment. Finally, we proved that SESN2 expression was highly associated with both phosphor‐AMPK and phosphor‐AKT expression in HCC tissues. In conclusion, SESN2‐induced activation of AKT and AMPK may serve as the novel mechanism underlying sorafenib primary resistance in HCC cells.

As one of the most common malignancy, HCC has aroused much attention to pre‐clinical and clinical studies in the past decades,^2^ partially because of high incidence of recurrence and metastasis after surgery as well as frequent resistance to current available therapeutic approaches, all of which commit to the poor prognosis of HCC. To be specific, although sorafenib effectively inhibited the HCC progression, resistance to this targeted therapy agent has obviously imposed limitations on its therapeutic efficacy. It is recognized that the long‐term administration with sorafenib in HCC patients and the constant stimulation by sorafenib in HCC cells give rise to acquired resistance to this systemic treatment agent and many studies have revealed that sorafenib acquired resistance was resulted from cancer stem cells,^37^ disabling of pro‐apoptotic signals,^38^ hypoxic microenvironment,^39^ up‐regulated autophagy,^7^, ^8^ and EMT.^9^, ^10^ Meanwhile, short‐term exposure to sorafenib yields decreased or even initially little therapeutic efficacy in some patients. It is potentially associated with genetic or molecular heterogeneity but the exact mechanism is far from understood.^40^ Therefore, it is of great clinical significance to further elucidate the molecular mechanism underlying sorafenib primary resistance.

It has been reported that the dysregulation of many endogenous signaling pathways was implicated in sorafenib resistance in HCC cells, though the upstream regulatory mechanisms need to be investigated. Among them, activation of cellular intrinsic pro‐survival pathway PI3K/AKT signaling, with multiple upstream regulators, has been covered in several studies about sorafenib resistance and it turned out to be involved in acquired sorafenib resistance. For instance, Wu et al found that adrenergic receptorβ2 activated AKT signaling to facilitate glucose metabolism reprogramming through mediating hypoxia‐inducible factor‐1α (HIF‐1α) stabilization, which resulted in acquired sorafenib resistance both in vivo and vitro.^11^, ^41^ In addition, Dietrich et al uncovered that dysregulation in the upstream mediator of PI3K/AKT, KRAS, led to sorafenib acquired resistance caused by loss of tumor suppressive microRNA‐622.^42^ Aside from this, we previously demonstrated that the occurrence of primary resistance after temporary sorafenib stimulation was attributed to activation of AKT signaling for facilitating cell survival,^43^ indicating that the activation of AKT was not only implicated in the acquired resistance of sorafenib treatment but also highly connected to sorafenib primary resistance, which is in accordance with previous studies.^13^, ^14^, ^15^ However, the upstream regulatory network of PI3K/AKT in sorafenib primary resistance is partially understood. It has already been confirmed that overexpression of miR‐494,^44^ as well as increased insulin‐like growth factor 1 receptor (IGF1R) expression^29^ was responsible for triggering AKT signaling activation to mediate sorafenib primary resistance in HCC. In addition, the combination of a histone deacetylase inhibitor valproic acid (VPA) with sorafenib was capable to inhibit AKT activation, thus helping to increase sensitivity to short‐term sorafenib exposure.^14^ Therefore, it may shed light on new insights to get over sorafenib resistance and prompt us to provide more focus on the upstream regulatory mechanisms of the activation of AKT. Our study revealed that elevation of stress‐inducible protein SESN2 expression participated in activating AKT signaling as a novel positive upstream regulator, which replenishes and expands the molecular network of sorafenib primary resistance in HCC and provides a potential target to increase sorafenib treatment efficacy.

The intracellular energy status sensor AMPK has been thought to promote cell survival under energy stress.^31^, ^35^ AMPK phosphorylation can be triggered by the excessive ATP consumption and suppressed ATP generation,^45^ so as to mediating intracellular energy stress response. In the condition of sorafenib treatment, AMPK has been revealed to be activated since the agent repressed mitochondrial respiration and consequently decreased ATP levels in cardiomyocytes, HCC cells, and breast cancer cells.^46^, ^47^, ^48^ Specifically, activation of AMPK plays a protective role against sorafenib‐induced de‐energization in hepatocholangiocarcinoma cells, revealing that AMPK depletion potentiated sorafenib treatment efficacy^31^ and AMPK activation contributed to sorafenib resistance. It has been widely known that AMPK phosphorylation is mediated by LKB1^45^ and Ca^2+^‐activated kinase, CaMKK2.^49^, ^50^ Nevertheless, endogenous mechanism modulating AMPK activation in sorafenib resistance is poorly understood. So far, in order to overcome sorafenib resistance and promote sorafenib therapeutic effectiveness, researchers have mainly dedicated to combining sorafenib with other agents associated with mediating AMPK activation like all‐trans retinoic acid (ATRA),^51^ 2‐deoxyglucose (2‐DG),^48^ metformin,^52^ capsaicin,^53^ aspirin^35^ etc. However, it is of more essence to reveal intrinsic regulatory mechanism of AMPK activation in sorafenib resistance. Extensively, in the present study, we proved that the elevated phosphor‐AMPK levels and the subsequent up‐regulated ATP levels were abrogated by SESN2 knockdown in HCC cells, implying that SESN2, as the critical upstream regulator was able to activate AMPK and promote ATP production, implicated in maintaining tumor cell survival. Therefore, SESN2 could be a potential target to overcome sorafenib primary resistance by regulating AMPK.

SESN2 plays a crucial role in cell survival and cellular metabolic rewiring.^20^, ^54^ Bensahra et al^21^ found that SESN2 protected cells from energetic stress‐induced death and Kumar et al^55^ reported that SESN2 raised the expression of peroxisome proliferator‐activated receptor γ coactivator‐1α (PGC‐1α) in HepG2 cells and facilitated survival of HCC cells after chemotherapeutic agents treatment. Moreover, SESN2 is found capable of inducing resistance to chemotherapeutic drugs through activating AKT signaling via the regulation of PTEN in human squamous cell carcinoma and melanoma cells.^13^ Identical to what previously studied, our study demonstrated that SESN2 was able to induce primary resistance to the targeted agent, sorafenib, in HCC cells via activating both AKT and AMPK, suggesting that SESN2 could be a novel target to limit HCC growth and to increase therapeutic sensitivity toward sorafenib. Whether alternative downstream mediators were involved in the effect of SESN2 on sorafenib primary resistance needs future investigations. Of note, the activation of autophagy has been regarded as a crucial mechanism of SESN2 to protect cells from energy stress and facilitate cellular survival.^20^ In addition, the alteration of cellular autophagy was one of the non‐negligible causes of sorafenib resistance.^2^, ^11^, ^27^, ^40^ Autophagy is a significant endogenous protective mechanism to maintain cellular homeostasis by degrading misfolded proteins and injured organelles. It has been unveiled that the receptor for advanced glycation end products (Rage) impaired cell autophagy via AMPK signaling and thus causing sorafenib primary resistance.^2^ In the meantime, SESN2 lifts up cell autophagy levels to hold cell survival through activating AMPK and inhibiting mTOR.^20^ Therefore, SESN2 may be involved in sorafenib resistance by regulating AMPK/mTOR signaling‐dependent cell autophagy, and it is worthwhile exploring the accurate mechanism with molecular regulators therein. According to previous studies, the treatment of sorafenib was capable to induce diverse stressful circumstances, including hypoxia^27^, ^56^ and oxidative stress,^13^, ^31^, ^57^ which was proved as critical triggers of SESN2 expression. In addition, sorafenib could potentiate the expressions of Nrf2 ,^58^, ^59^, ^60^ AP‐1,^61^, ^62^ and p53^63^, ^64^ that were evidently documented as the upstream transcriptional activators of SESN2. Therefore, the altered stressful tumor microenvironment and indicated changes of transcriptional regulators may possibly account for the increased SESN2 expression after sorafenib treatment in HCC, which need further investigations in the future.

Altogether, our results displayed that SESN2 was up‐regulated in HCC cells and tissues as a potential promoter for sorafenib primary resistance through simultaneously activating AKT and AMPK to restrain cell apoptosis. Targeting stress‐inducible protein SESN2 could be valuable therapeutic approach for overcoming sorafenib primary resistance in the future.

## CONFLICT OF INTEREST

The authors declare no conflict of interest.
