# Supplementary material for: Sestrin 2 confers primary resistance to sorafenib by simultaneously activating AKT and AMPK in hepatocellular carcinoma
Source: Cancer Med. 2018 Oct 11;7(11):5691–703. doi: 10.1002/cam4.1826 (PMC6247041; doi:10.1002/cam4.1826)

Supplementary Materials

F I G U R E S1 （**A** to **E**）The cell proliferation curves of each indicated HCC cell line. Sorafenib concentrations (C) were transformed into lgC. The experiments were performed in triplicate and the results are presented as Mean ±SD.

Supplementary Figures

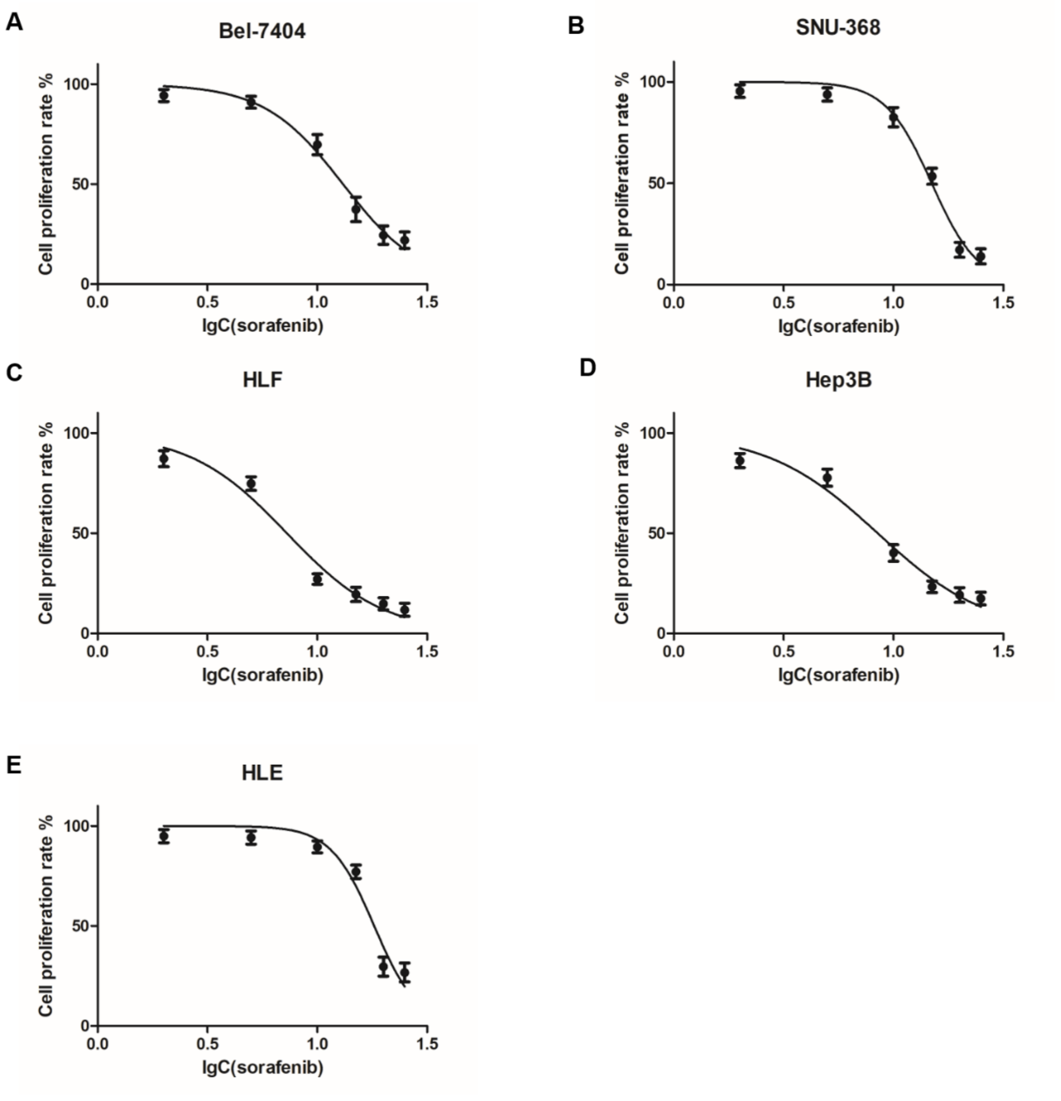

Supplement: Supplementary file 1 [file CAM4-7-5691-s001.pdf]
